# Supplementary material for: Analysis and Visualization of Confounders and Treatment Pathways Leading to Amputation and Non-Amputation in Peripheral Artery Disease Patients Using Sankey Diagrams: Enhancing Explainability
Source: Biomedicines. 2025 Jan 21;13(2):258. doi: 10.3390/biomedicines13020258 (PMC11851926; doi:10.3390/biomedicines13020258)
Supplement: Supplementary file 1 [file biomedicines-13-00258-s001.zip › biomedicines-3420873-supplementary.pdf]

biomedicines

MDPI

Supplementary Material

Analysis and Visualization of Confounders and Treatment Pathways Leading to Amputation and Non-Amputation in Peripheral Artery Disease Patients Using Sankey Diagrams: Enhancing Explainability

Rajashekar Korutla [1], Douglas Tedder [1], Kathryn Brogan [1], Marko Milosevic [1], Michael P. Wilczek [1], Naim Shehadeh [2], Nawar Shara [3,4], Elsie G. Ross [5] and Saeed Amal [1,6,*]

1 The Roux Institute, Northeastern University, Portland, ME 04101, USA; korutla.r@northeastern.edu (R.K.); tedder.do@northeastern.edu (D.T.); brogan.kat@northeastern.edu (K.B.); m.milosevic@northeastern.edu (M.M.); m.wilczek@northeastern.edu (M.P.W.)
2 The Russell Berrie Galilee Diabetes SPHERE, Azrieli Faculty of Medicine, Bar-Ilan University, Safad 5290002, Israel; naim.shehadeh@biu.ac.il
3 Center of Biostatistics, Informatics and Data Science at MedStar Health Research Institute (MHRI), Columbia, MD 21044, USA; nawar.shara@medstar.net
4 Health Data Science, Georgetown University (GU), Washington, DC 20007, USA
5 Department of Surgery, Division of Vascular Surgery, San Diego School of Medicine, University of California, La Jolla, San Diego, CA 92037, USA; e5ross@health.ucsd.edu
6 Department of Bioengineering, College of Engineering, Northeastern University, Boston, MA 02120, USA
* Correspondence: s.amal@northeastern.edu

Supplementary Material S1:

Table S1. Raw Counts of Unique Sequences in the PAD STARR Data.

Treatment Sequence	Amputation Count (%)	Non-Amputation Count (%)
Anti Platelet	4 (5.19%)	560 (10.22%)
Anti Platelet,Endovascular Revascularization	1 (1.3%)	59 (1.08%)
Anti Platelet,Endovascular Revascularization,Lipid Lowering	0 (0.0%)	16 (0.29%)
Anti Platelet,Exercise Therapy	0 (0.0%)	9 (0.16%)
Anti Platelet,Exercise Therapy,Lipid Lowering	1 (1.3%)	14 (0.26%)
Anti Platelet,Exercise Therapy,Lipid Lowering,Revascularization Surgery	0 (0.0%)	2 (0.04%)
Anti Platelet,Lipid Lowering	20 (25.97%)	1769 (32.29%)
Anti Platelet,Lipid Lowering,Endovascular Revascularization	6 (7.79%)	114 (2.08%)
Anti Platelet,Lipid Lowering,Endovascular Revascularization,Exercise Therapy	0 (0.0%)	2 (0.04%)
Anti Platelet,Lipid Lowering,Endovascular Revascularization,Revascularization Surgery	0 (0.0%)	13 (0.24%)
Anti Platelet,Lipid Lowering,Endovascular Revascularization,Revascularization Surgery,Exercise Therapy	0 (0.0%)	2 (0.04%)
Anti Platelet,Lipid Lowering,Exercise Therapy	1 (1.3%)	49 (0.89%)
Anti Platelet,Lipid Lowering,Exercise Therapy,Endovascular Revascularization	0 (0.0%)	5 (0.09%)
Anti Platelet,Lipid Lowering,Exercise Therapy,Revascularization Surgery	0 (0.0%)	2 (0.04%)

 https://doi.org/10.3390/biomedicines13020258

Anti Platelet,Lipid Lowering,Revascularization Surgery	1 (1.3%)	59 (1.08%)
Anti Platelet,Lipid Lowering,Revascularization Surgery,Endovascular Revascularization	0 (0.0%)	25 (0.46%)
Anti Platelet,Lipid Lowering,Revascularization Surgery,Exercise Therapy	0 (0.0%)	3 (0.05%)
Anti Platelet,Lipid Lowering,Smoking Cessation	0 (0.0%)	2 (0.04%)
Anti Platelet,Revascularization Surgery	1 (1.3%)	25 (0.46%)
Anti Platelet,Revascularization Surgery,Endovascular Revascularization	0 (0.0%)	6 (0.11%)
Anti Platelet,Revascularization Surgery,Endovascular Revascularization,Lipid Lowering	0 (0.0%)	2 (0.04%)
Anti Platelet,Revascularization Surgery,Lipid Lowering	4 (5.19%)	50 (0.91%)
Anti Platelet,Revascularization Surgery,Lipid Lowering,Endovascular Revascularization	0 (0.0%)	15 (0.27%)
Anti Platelet,Smoking Cessation,Lipid Lowering	0 (0.0%)	2 (0.04%)
Endovascular Revascularization	0 (0.0%)	28 (0.51%)
Endovascular Revascularization,Anti Platelet	0 (0.0%)	4 (0.07%)
Endovascular Revascularization,Lipid Lowering	1 (1.3%)	1 (0.02%)
Exercise Therapy	0 (0.0%)	5 (0.09%)
Exercise Therapy,Anti Platelet	0 (0.0%)	6 (0.11%)
Exercise Therapy,Anti Platelet,Lipid Lowering	0 (0.0%)	6 (0.11%)
Exercise Therapy,Lipid Lowering	0 (0.0%)	5 (0.09%)
Exercise Therapy,Lipid Lowering,Anti Platelet	0 (0.0%)	5 (0.09%)
Lipid Lowering	12 (15.58%)	925 (16.88%)
Lipid Lowering,Anti Platelet	17 (22.08%)	1320 (24.09%)
Lipid Lowering,Anti Platelet,Endovascular Revascularization	2 (2.6%)	83 (1.51%)
Lipid Lowering,Anti Platelet,Endovascular Revascularization,Revascularization Surgery	0 (0.0%)	7 (0.13%)
Lipid Lowering,Anti Platelet,Exercise Therapy	0 (0.0%)	41 (0.75%)
Lipid Lowering,Anti Platelet,Exercise Therapy,Endovascular Revascularization	0 (0.0%)	3 (0.05%)
Lipid Lowering,Anti Platelet,Revascularization Surgery	1 (1.3%)	59 (1.08%)
Lipid Lowering,Anti Platelet,Revascularization Surgery,Endovascular Revascularization	0 (0.0%)	21 (0.38%)
Lipid Lowering,Anti Platelet,Smoking Cessation	0 (0.0%)	2 (0.04%)
Lipid Lowering,Endovascular Revascularization	0 (0.0%)	10 (0.18%)
Lipid Lowering,Endovascular Revascularization,Anti Platelet	0 (0.0%)	5 (0.09%)
Lipid Lowering,Exercise Therapy	0 (0.0%)	6 (0.11%)
Lipid Lowering,Exercise Therapy,Anti Platelet	0 (0.0%)	18 (0.33%)
Lipid Lowering,Exercise Therapy,Anti Platelet,Endovascular Revascularization	0 (0.0%)	3 (0.05%)
Lipid Lowering,Revascularization Surgery	0 (0.0%)	7 (0.13%)
Lipid Lowering,Revascularization Surgery,Anti Platelet	0 (0.0%)	11 (0.2%)
Lipid Lowering,Revascularization Surgery,Anti Platelet,Endovascular Revascularization	0 (0.0%)	5 (0.09%)
Revascularization Surgery	0 (0.0%)	13 (0.24%)
Revascularization Surgery,Anti Platelet	0 (0.0%)	9 (0.16%)
Revascularization Surgery,Anti Platelet,Lipid Lowering	1 (1.3%)	15 (0.27%)

Revascularization Surgery,Anti Platelet,Lipid Lowering,Endovascular Revascularization	0 (0.0%)	2 (0.04%)
Revascularization Surgery,Lipid Lowering	1 (1.3%)	10 (0.18%)
Revascularization Surgery,Lipid Lowering,Anti Platelet	2 (2.6%)	27 (0.49%)
Revascularization Surgery,Lipid Lowering,Anti Platelet,Endovascular Revascularization	1 (1.3%)	8 (0.15%)
Revascularization Surgery,Lipid Lowering,Anti Platelet,Exercise Therapy	0 (0.0%)	2 (0.04%)
Smoking Cessation,Lipid Lowering,Anti Platelet	0 (0.0%)	2 (0.04%)

Table S2. Raw Counts of Unique Sequences in the PAD All of Us Data.

Treatment Sequence	Amputation Count (%)	Non-Amputation Count (%)
Anti Platelet	18 (10.23%)	948 (23.3%)
Anti Platelet,Endovascular Revascularization	3 (1.7%)	7 (0.17%)
Anti Platelet,Endovascular Revascularization,Lipid Lowering	0 (0.0%)	6 (0.15%)
Anti Platelet,Endovascular Revascularization,Revascularization Surgery	1 (0.57%)	4 (0.1%)
Anti Platelet,Lipid Lowering	25 (14.2%)	892 (21.93%)
Anti Platelet,Lipid Lowering,Endovascular Revascularization	5 (2.84%)	39 (0.96%)
Anti Platelet,Lipid Lowering,Endovascular Revascularization,Revascularization Surgery	0 (0.0%)	12 (0.29%)
Anti Platelet,Lipid Lowering,Exercise Therapy	0 (0.0%)	2 (0.05%)
Anti Platelet,Lipid Lowering,Revascularization Surgery	1 (0.57%)	9 (0.22%)
Anti Platelet,Lipid Lowering,Revascularization Surgery,Endovascular Revascularization	2 (1.14%)	2 (0.05%)
Anti Platelet,Revascularization Surgery	1 (0.57%)	4 (0.1%)
Anti Platelet,Revascularization Surgery,Endovascular Revascularization	0 (0.0%)	2 (0.05%)
Endovascular Revascularization	24 (13.64%)	187 (4.6%)
Endovascular Revascularization,Anti Platelet	2 (1.14%)	10 (0.25%)
Endovascular Revascularization,Anti Platelet,Lipid Lowering	2 (1.14%)	11 (0.27%)
Endovascular Revascularization,Anti Platelet,Lipid Lowering,Revascularization Surgery	1 (0.57%)	0 (0.0%)
Endovascular Revascularization,Lipid Lowering	1 (0.57%)	4 (0.1%)
Endovascular Revascularization,Lipid Lowering,Anti Platelet	2 (1.14%)	12 (0.29%)
Endovascular Revascularization,Revascularization Surgery	11 (6.25%)	33 (0.81%)
Endovascular Revascularization,Revascularization Surgery,Anti Platelet,Lipid Lowering	0 (0.0%)	2 (0.05%)
Exercise Therapy	0 (0.0%)	4 (0.1%)
Lipid Lowering	10 (5.68%)	586 (14.41%)

Supplementary Material S2 :

Table S3. Pathways in PAD with Hypertension cohort in the STARR Dataset, whose prevalence is higher in the amputation cohort than in the non-amputation cohort.

Amputation Pathways				
Stage 1 Treatment	Stage 2 Treatment	Stage 3 Treatment	Stage 4 Treatment	Amputation Rate (%)

Anti platelet	Revascularization surgery	Lipid lowering	-	7.14
Anti platelet	Lipid lowering	Endovascular revascularization	-	7.14
Revascularization surgery	Lipid lowering	-	-	1.79
Revascularization surgery	Lipid lowering	Anti platelet	Endovascular revascularization	1.79

Table S4. Pathways in PAD with Hypertension cohort in the STARR Dataset, whose prevalence is higher in the non-amputation cohort than in the amputation cohort.

**Non - Amputation Pathways**		
**Stage 1 Treatment**	**Stage 2 Treatment**	**Non-Amputation Rate (%)**
anti platelet	lipid lowering	33
lipid lowering	anti platelet	27
lipid lowering	-	15
Anti platelet	-	7.28

Table S5. Pathways in PAD with Hypertension cohort in the *All of Us* Dataset, whose prevalence is higher in the amputation cohort than in the non-amputation cohort.

**Amputation Pathways**			
**Stage 1 Treatment**	**Stage 2 Treatment**	**Stage 3 Treatment**	**Amputation Rate (%)**
Endovascular revascularization	-	-	13.29
Endovascular revascularization	Revascularization surgery	-	6.36
Lipid lowering	Anti platelet	Endovascular Revascularization	4.05
Lipid lowering	Anti platelet	Revascularization surgery	2.31

Table S6. Pathways in PAD with Hypertension cohort in the *All of Us* Dataset, whose prevalence is higher in the non-amputation cohort than in the amputation cohort.

**Non - Amputation Pathways**		
**Stage 1 Treatment**	**Stage 2 Treatment**	**Non-Amputation Rate (%)**

Lipid lowering	Anti platelet	28
Anti platelet	-	22
Anti platelet	Lipid lowering	22
Lipid lowering	-	14

Table S7. Pathways in PAD with Diabetes cohort in the STARR Dataset, whose prevalence is higher in the amputation cohort than in the non-amputation cohort.

**Amputation Pathways**			
**Stage 1 Treatment**	**Stage 2 Treatment**	**Stage 3 Treatment**	**Amputation Rate (%)**
Anti platelet	Lipid lowering	Endovascular revascularization	9.26
Anti platelet	Revascularization surgery	Lipid lowering	5.56
Lipid lowering	Anti platelet	Endovascular revascularization	3.7
Revascularization surgery	Lipid lowering	Anti platelet	3.7

Table S8. Pathways in PAD with Diabetes cohort in the STARR Dataset, whose prevalence is higher in the non-amputation cohort than in the amputation cohort.

**Non - Amputation Pathways**		
**Stage 1 Treatment**	**Stage 2 Treatment**	**Non-Amputation Rate (%)**
anti platelet	lipid lowering	33
lipid lowering	anti platelet	31
lipid lowering	-	15
Anti platelet	-	4.7

Table S9. Pathways in PAD with Diabetes cohort in the *All of Us* Dataset, whose prevalence is higher in the amputation cohort than in the non-amputation cohort.

**Amputation Pathways**			
**Stage 1 Treatment**	**Stage 2 Treatment**	**Stage 3 Treatment**	**Amputation Rate (%)**

Endovascular revascularization	-	-	13.75
Endovascular revascularization	Revascularization surgery	-	5.62
Lipid lowering	Anti platelet	Endovascular revascularization	3.7
Lipid lowering	antiplatelet	Anti platelet	1.8

Table S10. Pathways in PAD with Diabetes cohort in the *All of Us* Dataset, whose prevalence is higher in the non-amputation cohort than in the amputation cohort.

**Non - Amputation Pathways**		
**Stage 1 Treatment**	**Stage 2 Treatment**	**Non-Amputation Rate (%)**
Lipid lowering	Anti platelet	29
Anti platelet	Lipid lowering	23
Anti platelet	-	21
Lipid lowering	-	13

Table S11. Pathways in PAD with Heart Failure cohort in the STARR Dataset, whose prevalence is higher in the amputation cohort than in the non-amputation cohort.

**Amputation Pathways**			
**Stage 1 Treatment**	**Stage 2 Treatment**	**Stage 3 Treatment**	**Amputation Rate (%)**
Anti platelet	Lipid lowering	Endovascular revascularization	19.23
Lipid lowering	-	-	11.54
Lipid lowering	Anti platelet	Endovascular revascularization	7.69

Table S12. Pathways in PAD with Heart Failure cohort in the STARR Dataset, whose prevalence is higher in the non-amputation cohort than in the amputation cohort.

**Non - Amputation Pathways**		
**Stage 1 Treatment**	**Stage 2 Treatment**	**Non-Amputation Rate (%)**
anti platelet	lipid lowering	39
lipid lowering	anti platelet	28
Anti platelet	-	7.43

Table S13. Pathways in PAD with Heart Failure cohort in the *All of Us* Dataset, whose prevalence is higher in the amputation cohort than in the non-amputation cohort.

Amputation Pathways			
**Stage 1 Treatment**	**Stage 2 Treatment**	**Stage 3 Treatment**	**Amputation Rate (%)**
Endovascular revascularization	-	-	14.15
Endovascular revascularization	Revascularization surgery	-	7.55
Anti platelet	Lipid lowering	Endovascular revascularization	2.83

Table S14. Pathways in PAD with Heart Failure cohort in the *All of Us* Dataset, whose prevalence is higher in the non-amputation cohort than in the amputation cohort.

Non - Amputation Pathways		
**Stage 1 Treatment**	**Stage 2 Treatment**	**Non-Amputation Rate (%)**
Lipid lowering	Anti platelet	30
Anti platelet	Lipid lowering	25
Anti platelet	-	20

Table S15. Pathways in PAD with Cerebrovascular disease cohort in the *STARR* Dataset, whose prevalence is higher in the amputation cohort than in the non-amputation cohort.

Amputation Pathways			
**Stage 1 Treatment**	**Stage 2 Treatment**	**Stage 3 Treatment**	**Amputation Rate (%)**
Lipid lowering	Anti platelet	-	30.77
Anti platelet	Lipid lowering	Endovascular revascularization	11.54
Anti platelet	Revascularization surgery	Lipid lowering	7.69
Anti platelet	-	-	7.69

Table S16. Pathways in PAD with Cerebrovascular disease cohort in the STARR Dataset, whose prevalence is higher in the non-amputation cohort than in the amputation cohort.

Non - Amputation Pathways		
**Stage 1 Treatment**	**Stage 2 Treatment**	**Non-Amputation Rate (%)**
anti platelet	lipid lowering	35
lipid lowering	-	8.5

Table S17. Pathways in PAD with Cerebrovascular disease cohort in the *All of Us* Dataset, whose prevalence is higher in the amputation cohort than in the non-amputation cohort.

**Amputation Pathways**			
**Stage 1 Treatment**	**Stage 2 Treatment**	**Stage 3 Treatment**	**Amputation Rate (%)**
Endovascular revascularization	-	-	14.53
Endovascular revascularization	Revascularization surgery	-	6.84
Lipid lowering	Anti platelet	Endovascular revascularization	5.13

Table S18. Pathways in PAD with Cerebrovascular disease cohort in the *All of Us* Dataset, whose prevalence is higher in the non-amputation cohort than in the amputation cohort.

**Non - Amputation Pathways**		
**Stage 1 Treatment**	**Stage 2 Treatment**	**Non-Amputation Rate (%)**
lipid lowering	anti platelet	27
Anti platelet	Lipid lowering	23
Anti platelet	-	21
Lipid lowering	-	12

Table S19. Pathways in PAD with coronary artery disease cohort in the STARR Dataset, whose prevalence is higher in the amputation cohort than in the non-amputation cohort.

**Amputation Pathways**			
**Stage 1 Treatment**	**Stage 2 Treatment**	**Stage 3 Treatment**	**Amputation Rate (%)**
Anti platelet	Lipid lowering	Endovascular revascularization	13.95
Anti platelet	Revascularization surgery	Lipid lowering	4.65
Lipid lowering	Anti platelet	Endovascular revascularization	4.65
Revascularization surgery	Lipid lowering	Anti platelet	4.65

Table S20. Pathways in PAD with coronary artery disease cohort in the STARR Dataset, whose prevalence is higher in the non-amputation cohort than in the amputation cohort.

**Non - Amputation Pathways**		
**Stage 1 Treatment**	**Stage 2 Treatment**	**Non-Amputation Rate (%)**

anti platelet	lipid lowering	40
lipid lowering	anti platelet	30

Table S21. Pathways in PAD with Coronary artery disease cohort in the *All of Us* Dataset, whose prevalence is higher in the amputation cohort than in the non-amputation cohort.

Amputation Pathways			
**Stage 1 Treatment**	**Stage 2 Treatment**	**Stage 3 Treatment**	**Amputation Rate (%)**
Endovascular revascularization	-	-	15.38
Endovascular revascularization	Revascularization surgery	-	6.36
Lipid lowering	Anti platelet	Endovascular Revascularization	3.5
Anti platelet	Endovascular revascularization	-	2.1

Table S22. Pathways in PAD with Coronary artery disease cohort in the *All of Us* Dataset, whose prevalence is higher in the non-amputation cohort than in the amputation cohort.

Non - Amputation Pathways		
**Stage 1 Treatment**	**Stage 2 Treatment**	**Non-Amputation Rate (%)**
Lipid lowering	Anti platelet	29
Anti platelet	Lipid lowering	25
Anti platelet	-	20
Lipid lowering	-	11

Table S23. Pathways in PAD with Hyperlipidemia cohort in the STARR Dataset, whose prevalence is higher in the amputation cohort than in the non-amputation cohort.

Amputation Pathways			
**Stage 1 Treatment**	**Stage 2 Treatment**	**Stage 3 Treatment**	**Amputation Rate (%)**
Anti platelet	Lipid lowering	Endovascular revascularization	7.89
Anti platelet	Revascularization surgery	Lipid lowering	5.26
Anti platelet	-	-	3.95
Revascularization surgery	Lipid lowering	Anti platelet	2.63

Table S24. Pathways in PAD with Hyperlipidemia cohort in the STARR Dataset, whose prevalence is higher in the non-amputation cohort than in the amputation cohort.

**Non - Amputation Pathways**		
**Stage 1 Treatment**	**Stage 2 Treatment**	**Non-Amputation Rate (%)**
anti platelet	lipid lowering	35.80
lipid lowering	anti platelet	26.72

Table S25. Pathways in PAD with Hyperlipidemia cohort in the *All of Us* Dataset, whose prevalence is higher in the amputation cohort than in the non-amputation cohort.

**Amputation Pathways**			
**Stage 1 Treatment**	**Stage 2 Treatment**	**Stage 3 Treatment**	**Amputation Rate (%)**
Endovascular revascularization	-	-	14.12
Endovascular revascularization	Revascularization surgery	-	5.88
Lipid lowering	Anti platelet	Endovascular Revascularization	4.12
Anti platelet	Lipid lowering	Endovascular revascularization	2.94

Table S26. Pathways in PAD with Hyperlipidemia cohort in the *All of Us* Dataset, whose prevalence is higher in the non-amputation cohort than in the amputation cohort.

**Non - Amputation Pathways**		
**Stage 1 Treatment**	**Stage 2 Treatment**	**Non-Amputation Rate (%)**
Lipid lowering	Anti platelet	29
Anti platelet	Lipid lowering	23
Anti platelet	-	20
Lipid lowering	-	15

Table S27. Pathways in PAD with age > 50 cohort in the STARR Dataset, whose prevalence is higher in the amputation cohort than in the non-amputation cohort.

**Amputation Pathways**			
**Stage 1 Treatment**	**Stage 2 Treatment**	**Stage 3 Treatment**	**Amputation Rate (%)**
Lipid lowering	-	-	20.25
Anti platelet	Lipid lowering	Endovascular revascularization	7.59

Anti platelet	Revascularization surgery	Lipid lowering	5.06
Revascularization surgery	Lipid lowering	Anti platelet	2.53

Table S28. Pathways in PAD with age > 50 cohort in the STARR Dataset, whose prevalence is higher in the non-amputation cohort than in the amputation cohort.

**Non - Amputation Pathways**		
**Stage 1 Treatment**	**Stage 2 Treatment**	**Non-Amputation Rate (%)**
anti platelet	lipid lowering	32.43
lipid lowering	anti platelet	24.31

Table S29. Pathways in PAD with age > 50 cohort in the *All of Us* Dataset, whose prevalence is higher in the amputation cohort than in the non-amputation cohort.

**Amputation Pathways**			
**Stage 1 Treatment**	**Stage 2 Treatment**	**Stage 3 Treatment**	**Amputation Rate (%)**
Endovascular revascularization	-	-	17.14
Lipid lowering	Anti platelet	Endovascular revascularization	4.29
Anti platelet	Lipid lowering	Endovascular Revascularization	3.57
Revascularization surgery	-	-	2.86

Table S30. Pathways in PAD with age > 50 cohort in the *All of Us* Dataset, whose prevalence is higher in the non-amputation cohort than in the amputation cohort.

**Non - Amputation Pathways**		
**Stage 1 Treatment**	**Stage 2 Treatment**	**Non-Amputation Rate (%)**
Lipid lowering	Anti platelet	28.41
Anti platelet	Lipid lowering	21.05
Anti platelet	-	22.6
Lipid lowering	-	15.35

Table S31. Pathways in PAD with age <= 50 cohort in the STARR Dataset, whose prevalence is higher in the amputation cohort than in the non-amputation cohort.

Amputation Pathways		
**Stage 1 Treatment**	**Stage 2 Treatment**	**Amputation Rate (%)**
Anti platelet	Lipid lowering	66.67
Anti platelet	-	33.33

Table S32. Pathways in PAD with age <= 50 cohort in the STARR Dataset, whose prevalence is higher in the non-amputation cohort than in the amputation cohort.

Non - Amputation Pathways		
**Stage 1 Treatment**	**Stage 2 Treatment**	**Non-Amputation Rate (%)**
Lipid lowering	-	18.49
Lipid lowering	Anti platelet	17.36
Anti platelet	Endovascular revascularization	5.28

Table S33. Pathways in PAD with age <= 50 cohort in the *All of Us* Dataset, whose prevalence is higher in the amputation cohort than in the non-amputation cohort.

Amputation Pathways		
**Stage 1 Treatment**	**Stage 2 Treatment**	**Amputation Rate (%)**
Lipid lowering	Anti platelet	27.78
Anti platelet	Lipid lowering	25

Table S34. Pathways in PAD with age <= 50 cohort in the *All of Us* Dataset, whose prevalence is higher in the non-amputation cohort than in the amputation cohort.

Non - Amputation Pathways	
**Stage 1 Treatment**	**Non-Amputation Rate (%)**
Anti platelet	29
Lipid lowering	21.51

Table S35. Pathways in PAD with age > 65 cohort in the STARR Dataset, whose prevalence is higher in the amputation cohort than in the non-amputation cohort.

Amputation Pathways			
**Stage 1 Treatment**	**Stage 2 Treatment**	**Stage 3 Treatment**	**Amputation Rate (%)**
Lipid lowering	Anti platelet	-	31.37
Anti platelet	Lipid lowering	Endovascular revascularization	5.88

Table S36. Pathways in PAD with age > 65 cohort in the STARR Dataset, whose prevalence is higher in the non-amputation cohort than in the amputation cohort.

**Non - Amputation Pathways**		
**Stage 1 Treatment**	**Stage 2 Treatment**	**Non - Amputation Rate (%)**
Anti platelet	Lipid lowering	31.64
Lipid lowering	-	18.21
Anti platelet	-	9.2

Table S37. Pathways in PAD with age > 65 cohort in the *All of Us* Dataset, whose prevalence is higher in the amputation cohort than in the non-amputation cohort.

**Amputation Pathways**		
**Stage 1 Treatment**	**Stage 2 Treatment**	**Amputation Rate (%)**
Endovascular revascularization	-	24.07
Endovascular revascularization	Revascularization surgery	9.26
Revascularization surgery	-	5.56

Table S38. Pathways in PAD with age > 65 cohort in the *All of Us* Dataset, whose prevalence is higher in the non-amputation cohort than in the amputation cohort.

**Non - Amputation Pathways**		
**Stage 1 Treatment**	**Stage 2 Treatment**	**Non - Amputation Rate (%)**
Lipid lowering	Anti platelet	29.89
Anti platelet	-	21.73
Anti platelet	Lipid lowering	18.54
Lipid lowering	-	18.27

Table S39. Pathways in PAD with age <= 65 cohort in the STARR Dataset, whose prevalence is higher in the amputation cohort than in the non-amputation cohort.

**Amputation Pathways**			
**Stage 1 Treatment**	**Stage 2 Treatment**	**Stage 3 Treatment**	**Amputation Rate (%)**
Lipid lowering	-	-	24.14
Anti platelet	Lipid lowering	Endovascular revascularization	6.9
Anti platelet	Revascularization surgery	Lipid lowering	6.9

Table S40. Pathways in PAD with age <= 65 cohort in the STARR Dataset, whose prevalence is higher in the non-amputation cohort than in the amputation cohort.

**Non - Amputation Pathways**		
**Stage 1 Treatment**	**Stage 2 Treatment**	**Non - Amputation Rate (%)**
Anti platelet	Lipid lowering	31.86
Lipid lowering	Anti platelet	18.87
Anti platelet	-	14.34

Table S41. Pathways in PAD with age <= 65 cohort in the *All of Us* Dataset, whose prevalence is higher in the amputation cohort than in the non-amputation cohort.

**Amputation Pathways**			
**Stage 1 Treatment**	**Stage 2 Treatment**	**Stage 3 Treatment**	**Amputation Rate (%)**
Endovascular revascularization	-	-	10.92
Endovascular revascularization	Revascularization surgery	-	5.04
Lipid lowering	Anti platelet	Endovascular revascularization	4.2

Table S42. Pathways in PAD with age <= 65 cohort in the *All of Us* Dataset, whose prevalence is higher in the non-amputation cohort than in the amputation cohort.

**Non - Amputation Pathways**		
**Stage 1 Treatment**	**Stage 2 Treatment**	**Non - Amputation Rate (%)**
Lipid lowering	Anti platelet	24.92
Anti platelet	-	24.48
Anti platelet	Lipid lowering	21.1
Lipid lowering	-	18.51

Table S43. Pathways in PAD with age <= 80 cohort in the STARR Dataset, whose prevalence is higher in the amputation cohort than in the non-amputation cohort.

**Amputation Pathways**			
**Stage 1 Treatment**	**Stage 2 Treatment**	**Stage 3 Treatment**	**Amputation Rate (%)**
Lipid lowering	-	-	18
Anti platelet	Lipid lowering	Endovascular revascularization	6.56

Anti platelet	Revascularization surgery	Lipid lowering	6.56
Revascularization surgery	Lipid lowering	Anti platelet	3.28

Table S44. Pathways in PAD with age <= 80 cohort in the STARR Dataset, whose prevalence is higher in the non-amputation cohort than in the amputation cohort.

**Non - Amputation Pathways**		
**Stage 1 Treatment**	**Stage 2 Treatment**	**Non - Amputation Rate (%)**
Anti platelet	Lipid lowering	31.94
Lipid lowering	Anti platelet	22.74
Anti platelet	-	11.08

Table S45. Pathways in PAD with age <= 80 cohort in the *All of Us* Dataset, whose prevalence is higher in the amputation cohort than in the non-amputation cohort.

**Amputation Pathways**			
**Stage 1 Treatment**	**Stage 2 Treatment**	**Stage 3 Treatment**	**Amputation Rate (%)**
Endovascular revascularization	-	-	13.71
Endovascular revascularization	Revascularization surgery	-	5.71
Lipid lowering	Anti platelet	Endovascular revascularization	4.0

Table S46. Pathways in PAD with age <= 80 cohort in the *All of Us* Dataset, whose prevalence is higher in the non-amputation cohort than in the amputation cohort.

**Non - Amputation Pathways**		
**Stage 1 Treatment**	**Stage 2 Treatment**	**Non - Amputation Rate (%)**
Lipid lowering	Anti platelet	27.15
Anti platelet	-	23.18
Anti platelet	Lipid lowering	21.99
Lipid lowering	-	14.78

Table S47. Pathways in PAD with age > 80 cohort in the STARR Dataset, whose prevalence is higher in the amputation cohort than in the non-amputation cohort.

**Amputation Pathways**

Stage 1 Treatment	Stage 2 Treatment	Stage 3 Treatment	Amputation Rate (%)
Lipid lowering	Anti platelet	-	42.11
Anti platelet	Lipid lowering	Endovascular revascularization	10.53

Table S48. Pathways in PAD with age > 80 cohort in the STARR Dataset, whose prevalence is higher in the non-amputation cohort than in the amputation cohort.

**Non - Amputation Pathways**		
**Stage 1 Treatment**	**Stage 2 Treatment**	**Non - Amputation Rate (%)**
Anti platelet	Lipid lowering	30.98
Lipid lowering	-	22.75

Table S49. Pathways in PAD with age > 80 cohort in the *All of Us* Dataset, whose prevalence is higher in the amputation cohort than in the non-amputation cohort.

**Amputation Pathways**		
**Stage 1 Treatment**	**Stage 2 Treatment**	**Amputation Rate (%)**
Endovascular revascularization	Revascularization surgery	50
Revascularization surgery	-	50

Table S50. Pathways in PAD with age > 80 cohort in the *All of Us* Dataset, whose prevalence is higher in the non-amputation cohort than in the amputation cohort.

**Non - Amputation Pathways**		
**Stage 1 Treatment**	**Stage 2 Treatment**	**Non - Amputation Rate (%)**
Lipid lowering	Anti platelet	30.52
Lipid lowering	-	23

Table S51. Pathways in PAD filtered for patients who reported their race as ‘white’ cohort in the STARR Dataset, whose prevalence is higher in the amputation cohort than in the non-amputation cohort.

**Amputation Pathways**			
**Stage 1 Treatment**	**Stage 2 Treatment**	**Stage 3 Treatment**	**Amputation Rate (%)**
Anti platelet	Lipid lowering	Endovascular revascularization	7.69
Anti platelet	Revascularization surgery	Lipid lowering	7.69
Revascularization surgery	Lipid lowering	Anti platelet	5.13

Table S52. Pathways in PAD filtered for patients who reported their race as 'white' cohort in the STARR Dataset, whose prevalence is higher in the non-amputation cohort than in the amputation cohort.

**Non - Amputation Pathways**		
**Stage 1 Treatment**	**Stage 2 Treatment**	**Non - Amputation Rate (%)**
Anti Platelet	Lipid lowering	31.84
Lipid lowering	Anti platelet	23.15
Lipid lowering	-	15.38
Anti platelet	-	10.26

Table S53. Pathways in PAD filtered for patients who reported their race as 'white' cohort in the *All of Us* Dataset, whose prevalence is higher in the amputation cohort than in the non-amputation cohort.

**Amputation Pathways**			
**Stage 1 Treatment**	**Stage 2 Treatment**	**Stage 3 Treatment**	**Amputation Rate (%)**
Endovascular revascularization	-	-	15.38
Endovascular revascularization	Revascularization surgery	-	8.97
Lipid lowering	Anti platelet	Endovascular revascularization	3.85

Table S54. Pathways in PAD filtered for patients who reported their race as 'white' cohort in the *All of Us* Dataset, whose prevalence is higher in the non-amputation cohort than in the amputation cohort.

**Non - Amputation Pathways**		
**Stage 1 Treatment**	**Stage 2 Treatment**	**Non - Amputation Rate (%)**
Lipid lowering	Anti platelet	29.59
Anti platelet	-	22.61
Anti platelet	Lipid lowering	20.72
Lipid lowering	-	15.80

Table S55. Pathways in PAD patients who reported their race as 'black' cohort in the STARR Dataset, whose prevalence is higher in the amputation cohort than in the non-amputation cohort.

**Amputation Pathways**			
**Stage 1 Treatment**	**Stage 2 Treatment**	**Stage 3 Treatment**	**Amputation Rate (%)**
Lipid lowering	-	-	21.43
Anti platelet	Lipid lowering	Endovascular revascularization	14.29

Table S56. Pathways in PAD patients who reported their race as 'black' cohort in the STARR Dataset, whose prevalence is higher in the non-amputation cohort than in the amputation cohort.

**Non - Amputation Pathways**		
**Stage 1 Treatment**	**Stage 2 Treatment**	**Non - Amputation Rate (%)**
Lipid lowering	Anti platelet	31.65
Anti platelet	Lipid lowering	29.29
Anti platelet	-	10.77

Table S57. Pathways in PAD patients who reported their race as 'black' cohort in the *All of Us* Dataset, whose prevalence is higher in the amputation cohort than in the non-amputation cohort.

**Amputation Pathways**				
**Stage 1 Treatment**	**Stage 2 Treatment**	**Stage 3 Treatment**	**Stage 4 Treatment**	**Amputation Rate (%)**
Endovascular revascularization	-	-	-	7.46
Lipid lowering	Anti platelet	Endovascular revascularization	-	4.48
Lipid lowering	Anti platelet	Endovascular revascularization	Revascularization surgery	4.48

Table S58. Pathways in PAD patients who reported their race as 'black' cohort in the *All of Us* Dataset, whose prevalence is higher in the non-amputation cohort than in the amputation cohort.

**Non - Amputation Pathways**		
**Stage 1 Treatment**	**Stage 2 Treatment**	**Non - Amputation Rate (%)**
Anti platelet	-	27.65
Anti platelet	Lipid lowering	24.18
Lipid lowering	Anti platelet	22.70
Lipid lowering	-	11.69

Table S59. Pathways in PAD patients who reported their race as 'Asian' cohort in the STARR Dataset, whose prevalence is higher in the amputation cohort than in the non-amputation cohort.

**Amputation Pathways**		
**Stage 1 Treatment**	**Stage 2 Treatment**	**Amputation Rate (%)**
Lipid lowering	Anti platelet	50

Anti platelet	Lipid lowering	50

Table S60. Pathways in PAD patients who reported their race as 'Asian' cohort in the STARR Dataset, whose prevalence is higher in the non-amputation cohort than in the amputation cohort.

**Non Amputation Pathways**	
**Stage 1 Treatment**	**Amputation Rate (%)**
Lipid lowering	16.76
Anti platelet	7.67

Table S61. Pathways in PAD patients who reported their race as 'Asian' cohort in the *All of Us* Dataset, whose prevalence is higher in the amputation cohort than in the non-amputation cohort.

**Amputation Pathways**	
**Stage 1 Treatment**	**Amputation Rate (%)**
Endovascular revascularization	100

Table S62. Pathways in PAD patients who reported their race as 'Asian' cohort in the *All of Us* Dataset, whose prevalence is higher in the non-amputation cohort than in the amputation cohort.

**Non - Amputation Pathways**		
**Stage 1 Treatment**	**Stage 2 Treatment**	**Non - Amputation Rate (%)**
Lipid lowering	Anti platelet	33.33
Anti platelet	Lipid lowering	33.33
Lipid lowering	-	23.08

Table S63. Pathways in PAD patients who reported their gender as 'male' cohort in the STARR Dataset, whose prevalence is higher in the amputation cohort than in the non-amputation cohort.

**Amputation Pathways**			
**Stage 1 Treatment**	**Stage 2 Treatment**	**Stage 3 Treatment**	**Amputation Rate (%)**
Anti platelet	Lipid lowering	Endovascular revascularization	8.93
Anti platelet	Revascularization surgery	Lipid lowering	7.14

Table S64. Pathways in PAD patients who reported their gender as 'male' cohort in the STARR Dataset, whose prevalence is higher in the non-amputation cohort than in the amputation cohort.

**Non - Amputation Pathways**		
**Stage 1 Treatment**	**Stage 2 Treatment**	**Non - Amputation Rate (%)**

Anti platelet	Lipid lowering	33.45
Lipid lowering	Anti platelet	25.32
Lipid lowering	-	16.40

Table S65. Pathways in PAD patients who reported their gender as 'male' cohort in the *All of Us* Dataset, whose prevalence is higher in the amputation cohort than in the non-amputation cohort.

**Amputation Pathways**		
**Stage 1 Treatment**	**Stage 2 Treatment**	**Amputation Rate (%)**
Endovascular revascularization	-	14.29
Endovascular revascularization	Revascularization surgery	7.62

Table S66. Pathways in PAD patients who reported their gender as 'male' cohort in the *All of Us* Dataset, whose prevalence is higher in the non-amputation cohort than in the amputation cohort.

**Non - Amputation Pathways**		
**Stage 1 Treatment**	**Stage 2 Treatment**	**Non - Amputation Rate (%)**
Lipid lowering	Anti platelet	29.89
Anti platelet	Lipid lowering	21.33
Anti platelet	-	21.44

Table S67. Pathways in PAD patients who reported their gender as 'Female' cohort in the STARR Dataset, whose prevalence is higher in the amputation cohort than in the non-amputation cohort.

**Amputation Pathways**			
**Stage 1 Treatment**	**Stage 2 Treatment**	**Stage 3 Treatment**	**Amputation Rate (%)**
Lipid lowering	Anti platelet	-	47.62
Anti platelet	Exercise therapy	Lipid lowering	4.76
Endovascular revascularization	Lipid lowering	-	4.76

Table S68. Pathways in PAD patients who reported their gender as 'Female' cohort in the STARR Dataset, whose prevalence is higher in the non-amputation cohort than in the amputation cohort.

**Non - Amputation Pathways**		
**Stage 1 Treatment**	**Stage 2 Treatment**	**Non - Amputation Rate (%)**
Anti platelet	Lipid lowering	30.86
Lipid lowering	-	17.77

Anti platelet	-	12.71

Table S69. Pathways in PAD patients who reported their gender as 'Female' cohort in the *All of Us* Dataset, whose prevalence is higher in the amputation cohort than in the non-amputation cohort.

**Amputation Pathways**			
**Stage 1 Treatment**	**Stage 2 Treatment**	**Amputation Rate (%)**	
Lipid lowering	Anti platelet	27.27	
Endovascular revascularization	-	13.68	

Table S70. Pathways in PAD patients who reported their gender as 'Female' cohort in the *All of Us* Dataset, whose prevalence is higher in the non-amputation cohort than in the amputation cohort.

**Non - Amputation Pathways**		
**Stage 1 Treatment**	**Stage 2 Treatment**	**Non - Amputation Rate (%)**
Anti platelet	-	25.06
Anti platelet	Lipid lowering	22.78
Lipid lowering	-	16.48

Table S71. Pathways in PAD patients who reportedly smoke cohort in the STARR Dataset, whose prevalence is higher in the amputation cohort than in the non-amputation cohort.

**Amputation Pathways**			
**Stage 1 Treatment**	**Stage 2 Treatment**	**Stage 3 Treatment**	**Amputation Rate (%)**
Anti platelet	Lipid lowering	Endovascular revascularization	8.33
Anti platelet	Revascularization surgery	Lipid lowering	5.56

Table S72. Pathways in PAD patients who reportedly smoke cohort in the STARR Dataset, whose prevalence is higher in the non-amputation cohort than in the amputation cohort.

**Non - Amputation Pathways**		
**Stage 1 Treatment**	**Stage 2 Treatment**	**Non - Amputation Rate (%)**
Anti platelet	Lipid lowering	32.14
Lipid lowering	Anti platelet	24.62
Lipid lowering	-	16.45
Anti platelet	-	9.64

Table S73. Pathways in PAD patients who reportedly smoke cohort in the *All of Us* Dataset, whose prevalence is higher in the amputation cohort than in the non-amputation cohort.

**Amputation Pathways**			
**Stage 1 Treatment**	**Stage 2 Treatment**	**Stage 3 Treatment**	**Amputation Rate (%)**
Endovascular revascularization	-	-	11.54
Endovascular revascularization	Revascularization surgery	-	6.41
Anti platelet	Lipid lowering	Endovascular revascularization	3.85

Table S74. Pathways in PAD patients who reportedly smoke cohort in the All of Us Dataset, whose prevalence is higher in the non-amputation cohort than in the amputation cohort.

**Non - Amputation Pathways**		
**Stage 1 Treatment**	**Stage 2 Treatment**	**Non - Amputation Rate (%)**
Lipid lowering	Anti platelet	24.90
Anti platelet	-	22.78
Anti platelet	Lipid lowering	21
